# Trends in the relative prevalence of genital ulcer disease pathogens and association with HIV infection in Johannesburg, South Africa, 2007–2015

**DOI:** 10.1371/journal.pone.0194125

**Published:** 2018-04-04

**Authors:** Ranmini S. Kularatne, Etienne E. Muller, Dumisile V. Maseko, Tendesayi Kufa-Chakezha, David A. Lewis

**Affiliations:** 1 Centre for HIV & STIs, National Institute for Communicable Diseases, Johannesburg, South Africa; 2 Department of Clinical Microbiology & Infectious Diseases, Faculty of Health Sciences, University of the Witwatersrand, Johannesburg, South Africa; 3 Marie Bashir Institute for Infectious Diseases and Biosecurity, University of Sydney, Sydney, Australia; 4 Western Sydney Sexual Health Centre, Western Sydney Local Health District, Sydney, Australia; University of Ottawa, CANADA

## Abstract

**Background:**

In South Africa, treatment of genital ulcer disease (GUD) occurs in the context of syndromic management. GUD aetiological studies have been conducted in Johannesburg since 2007. We report on GUD pathogen prevalence, sero-prevalence of STI co-infections and aetiological trends among GUD patients presenting to a community-based primary healthcare facility in Johannesburg over a 9-year period.

**Methods and findings:**

GUD surveys were conducted from January to April each year. Consecutive genital ulcers were sampled from consenting adults. Swab-extracted DNA was tested by multiplex real-time PCR assays for herpes simplex virus (HSV), *Treponema pallidum* (TP), *Haemophilus ducreyi* (HD) and *Chlamydia trachomatis* (CT). HSV-positive DNA extracts were further subtyped into HSV-1 and HSV-2 using a commercial PCR assay; CT-positive extracts were tested with an in-house PCR assay specific for serovars L1-L3 (lymphogranuloma venereum). Sera were tested for HIV, HSV-2, and syphilis co-infections. Giemsa-stained ulcer smears were screened for *Klebsiella granulomatis* by microscopy. Data were analysed with STATA^TM^ version 14. Of 771 GUD specimens, 503 (65.2%) had a detectable pathogen: HSV 468 (60.7%); TP 30 (3.9%); CT L1-3 7 (0.9%); HD 4 (0.5%). No aetiological agents were detected in 270 (34.8%) ulcer specimens. Seroprevalence rates were as follows: HIV 61.7%; HSV-2 80.2% and syphilis 5.8%. There was a strong association between GUD pathogen detection and HIV seropositivity (p < 0.001); 68% of cases caused by HSV were co-infected with HIV. There was a significant decline in the relative prevalence of ulcer-derived HSV over time, predominantly from 2013–2015 (p-value for trend = 0.023); and a trend towards a decrease in the HIV seropositivity rate (p-value for trend = 0.209).

**Conclusions:**

HSV remains the leading cause of pathogen-detectable GUD in South Africa. The prevalence of HIV co-infection among GUD patients is high, underlining the importance of linkage to universal HIV testing and treatment in primary healthcare settings.

## Introduction

Genital ulcer disease (GUD) has been recognized as a significant risk factor in the acquisition and transmission of human immunodeficiency virus (HIV) infection in Africa.[1–4] In South Africa, the HIV infection prevalence among adults in the reproductive age group was estimated to be 19.2% in 2015.[5] Knowledge of the major aetiologies of GUD and appropriate management is a public health priority.

Treatment of genital ulcer disease (GUD) in South Africa occurs in the context of syndromic management at primary healthcare level with the use of standard treatment guidelines.[6] Syndromic management of STIs ensures that the major sexually transmitted pathogens responsible for the condition are correctly treated. It is recommended by the WHO as a pragmatic strategy in areas that lack the requisite resources or diagnostic facilities for universal aetiological testing.[7] The epidemiology of GUD in South Africa underwent a paradigm shift in the early 2000s at the onset of the HIV epidemic. Chancroid, the predominant cause of GUD in the latter part of the 20^th^ century, was virtually eliminated by the addition of macrolide therapy to the GUD syndromic management algorithm.[8] In 2008, acyclovir was added to the genital ulcer syndrome treatment algorithm on the grounds that aetiological surveillance highlighted that herpes simplex virus (HSV) was the predominant genital ulcer-associated pathogen, and an in-country randomized clinical trial reported benefit of acyclovir treatment on ulcer healing.[9, 10] These important shifts in the relative prevalence of HSV were observed in an era characterized by maturation of the HIV epidemic, improved public health management of GUD and public health campaigns to reduce sexual risk-taking and increase HIV testing behaviours.

The Centre for HIV and STIs at the National Institute for Communicable Diseases in Johannesburg, South Africa, conducts aetiological surveillance for major sexually transmitted infection (STI) syndromes, including GUD, across the nine provinces of the country, in order to validate existing syndromic management guidelines. GUD aetiological studies have been undertaken on an annual basis at the same clinical site in Johannesburg, the capital of the most populous province of the country, since 2007.

We report on GUD pathogen prevalence, sero-prevalence of STI co-infections including HIV and aetiological trends among GUD patients presenting to a community-based primary healthcare facility in Johannesburg over a 9-year period.

## Materials and methods

### Study setting and participant enrolment

GUD surveys were conducted at the Alexandra Health Centre (AHC) in Johannesburg from January to April each year between 2007 and 2015. The AHC offers a variety of community oriented primary healthcare services, including STI syndromic treatment, HIV counselling and testing, and prevention services such as condom promotion and distribution. Patient inclusion criteria included consecutive adult males and females 18 years or older presenting with a new episode of GUD that was still suitable for diagnostic assessment (i.e. not healed). Consecutive adult patients presenting with GUD were invited to participate in the survey by trained professional nurses. Patients were enrolled only once for any given STI syndrome (e.g. GUD) in a given four-month collection period. They were included in the survey more than once only if they presented with a different STI syndrome within the four-month surveillance period. Each patient was given a patient information sheet to read, and an opportunity to ask the nurse questions, following which they could consent in written format to participation in the survey and the collection of biological specimens.

### Data and specimen collection

Following written informed consent, demographic and behavioural information, delinked from personal identifiers, was collected using a short nurse-administered questionnaire. The questionnaire recorded key demographic, STI clinical and sexual behaviour variables such as patient age, gender, ethnicity (defined as African, Mixed race, Indian, White or Other), sexual orientation (defined as heterosexual, bisexual, homosexual), history of STI syndromes in the past one year (including GUD), as well as other STI syndromes diagnosed in the patient at presentation.

Dacron swabs were used to collect material from the base and edges of ulcerative lesions. Ulcer exudate was rolled over the centre of a labelled glass slide which was allowed to air dry. The swab was then placed in a sterile container for use in molecular testing. A 10ml venous blood specimen was collected from each patient for serological testing. Clinical specimens were linked to the respective questionnaires by means of survey numbers, and all were devoid of any patient identifying information. Swab specimens were transported on ice on the date of collection to the STI Reference laboratory at the National Institute for Communicable Diseases in Johannesburg.

All patients were managed according to routine standard of care practices at the health centre, which included antimicrobial treatment according to national syndromic management flowcharts.[6]

### Laboratory procedures

DNA was extracted from swabs using two automated DNA extractors (X-tractor Gene and QIAxtractor platforms, Qiagen, Hilden, Germany). Swab-extracted DNA was tested by a validated in-house real-time multiplex polymerase chain reaction (PCR) assay for herpes simplex virus (HSV), *Treponema pallidum* (TP), *Haemophilus ducreyi* (HD) and *Chlamydia trachomatis* (CT), as described previously.[11] A commercial type-specific real-time PCR was used to subtype HSV positive DNA extracts into HSV-1 and HSV-2 (Sacace Biotechnologies, Como, Italy). An in-house real-time PCR was used to determine whether *C*. *trachomatis*, when detected, represented a serovar causing lymphogranuloma venereum (CT L1-3)[12]. All real-time PCR assays were performed on the RotorGene real-time PCR system (Qiagen, Hilden, Germany). Serum specimens were screened for HIV using two rapid immunochromatographic assays (Unigold™ Trinity Biotech, Trinity Biotech PLC, Wicklow, Ireland; Alere Determine™, Alere Medical Co. Ltd, Chiba, Japan) and for HSV-2 infection using the Focus HerpeSelect® 2 ELISA IgG assay (Focus Diagnostics, Cypress, CA, USA). Syphilis co-infection was detected using a rapid plasma reagin Immutrep® RPR assay (Omega Diagnostics Ltd, Alva, UK). Ulcer smears were fixed with methanol prior to staining with Giemsa stain. Stained slides were screened microscopically for the presence of Donovan bodies that are pathognomic of granuloma inguinale.

### Data management and statistical analysis

Data from completed clinical questionnaires and laboratory results were entered into a survey specific Access database [Microsoft, Seattle, WA, USA]. Data were exported into STATA 14 [Stata Corporation, College Station, TX, USA] for analysis. The enrolled participants were described using frequencies and proportions for categorical data, and medians and interquartile ranges (IQRs) for continuous variables. The relative prevalence of the different STI pathogens and seroprevalence of HIV, HSV-2 and RPR were determined as proportions with Wilson’s 95% confidence intervals around them. Pearson’s Chi square and Fischer’s exact tests were used to test for associations in the prevalence of ulcer pathogens by year and HIV serostatus, with the level of significance defined as p < 0.05. Logistic regression and likelihood ratio test were used to determine trends and associations in the relative prevalence of ulcer pathogens and HIV serostatus over time.

### Ethical considerations

Ethical approval for the study was granted by the Human Research Ethics Committee (Medical) of the University of the Witwatersrand, Johannesburg, South Africa (certificate numbers M120365 and M131129).

## Results

### Patient characteristics

A total of 771 patients presenting with GUD were enrolled between 2007 and 2015 (Table 1). Demographic and clinical data were available for a subset of participants (Table 2). Complete demographic and clinical data were missing for 90 patients, most of whom were enrolled in 2007 (75/90 overall and 75/76 enrolled in 2007), due to operational challenges experienced with the database during this particular year. Most patients were male (414/681; 60.8%). The median age of patients was 29 years (n = 672; IQR 25–35 years). Nearly 52% (352/681) of patients had presented with an STI syndrome within the preceding one-year period; in nearly 40% (258/681), this represented a previous episode of GUD. A concomitant STI syndrome was present at enrolment in 32.0% (218/681) of patients; these included Vaginal Discharge Syndrome (VDS) in 70.2% (153/218) of women and Male Urethritis Syndrome (MUS) in 29.8% (65/218) of men. Information on self-reported sexual orientation was available for 68.6% (529/771) patients; of these, 99.8% (528/529) described themselves as heterosexual.

**Table 1:** Number and proportion of participants enrolled by year.

Year of surveillance	Number of participants	% of Total participants enrolled (n = 771)
2007	76	9.9
2008	144	18.7
2009	100	13.0
2010	142	18.4
2011	68	8.8
2012	87	11.3
2013	48	6.2
2014	58	7.5
2015	48	6.2

**Table 2:** Demographic and clinical characteristics of patients presenting with GUD to a community healthcare centre in Johannesburg, 2007–2015.

Variable	N	
Age in years (median, IQR*)	672	29 (25–34.5)*
Males (n, %)	681	414 (60.8)
Heterosexual orientation (n, %)	529	528 (99.8)
Previous STI (n, %)	681	352 (51.7)
Previous GUD (n, %)	681	258 (37.9)
Concominant STI Syndrome (n, %)	681	218 (32.0)
Concominant VDS**	218	153 (70.0)
Concominant MUS***	218	65 (29.8)

*IQR = interquartile range

** VDS = Vaginal Discharge Syndrome

***MUS = Male Urethritis Syndrome

### Prevalence of GUD pathogens

**Fig 1:**
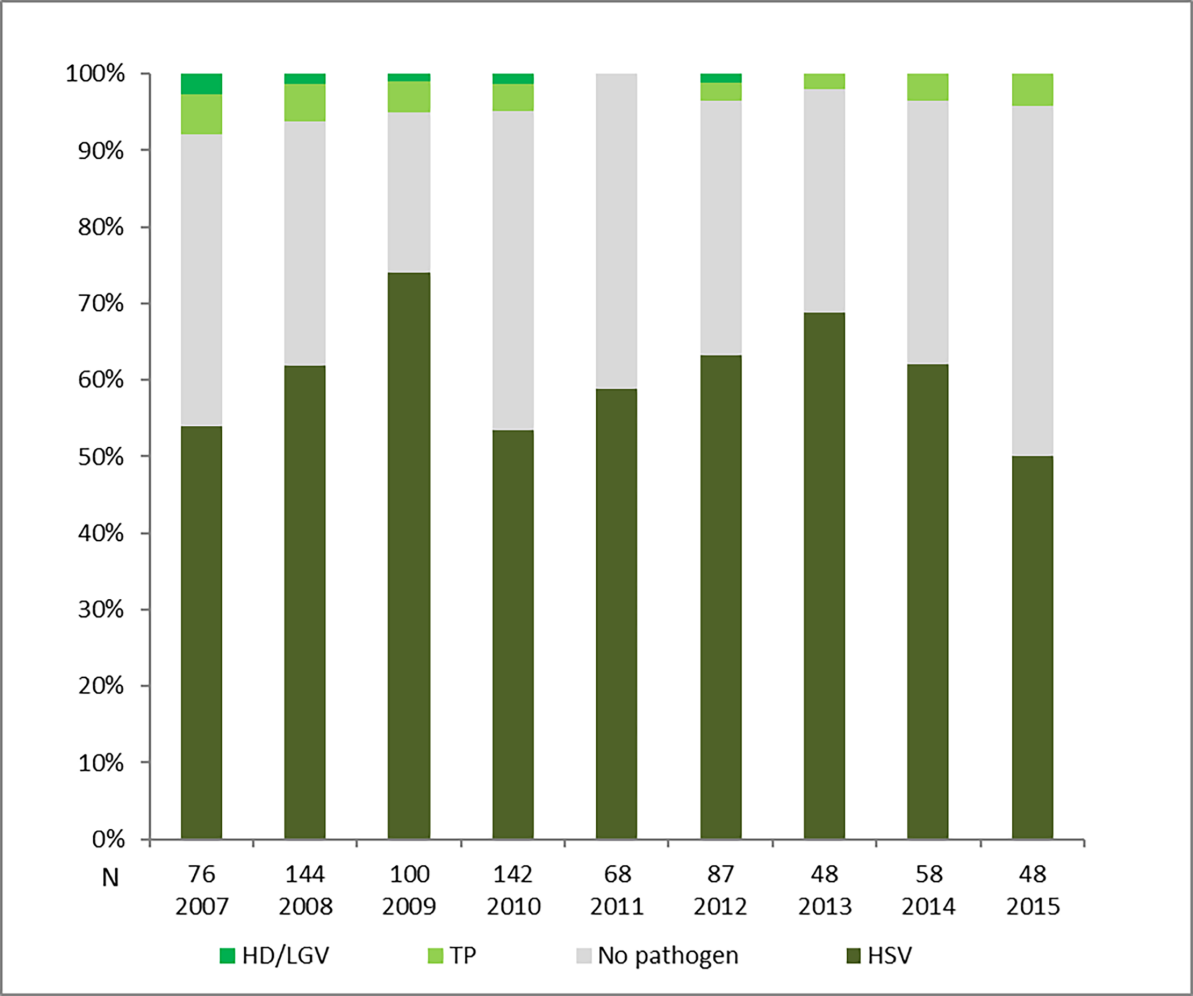
Relative prevalence of GUD aetiologies by year of enrolment, 2007–2015 (n = 771).

**Fig 2:**
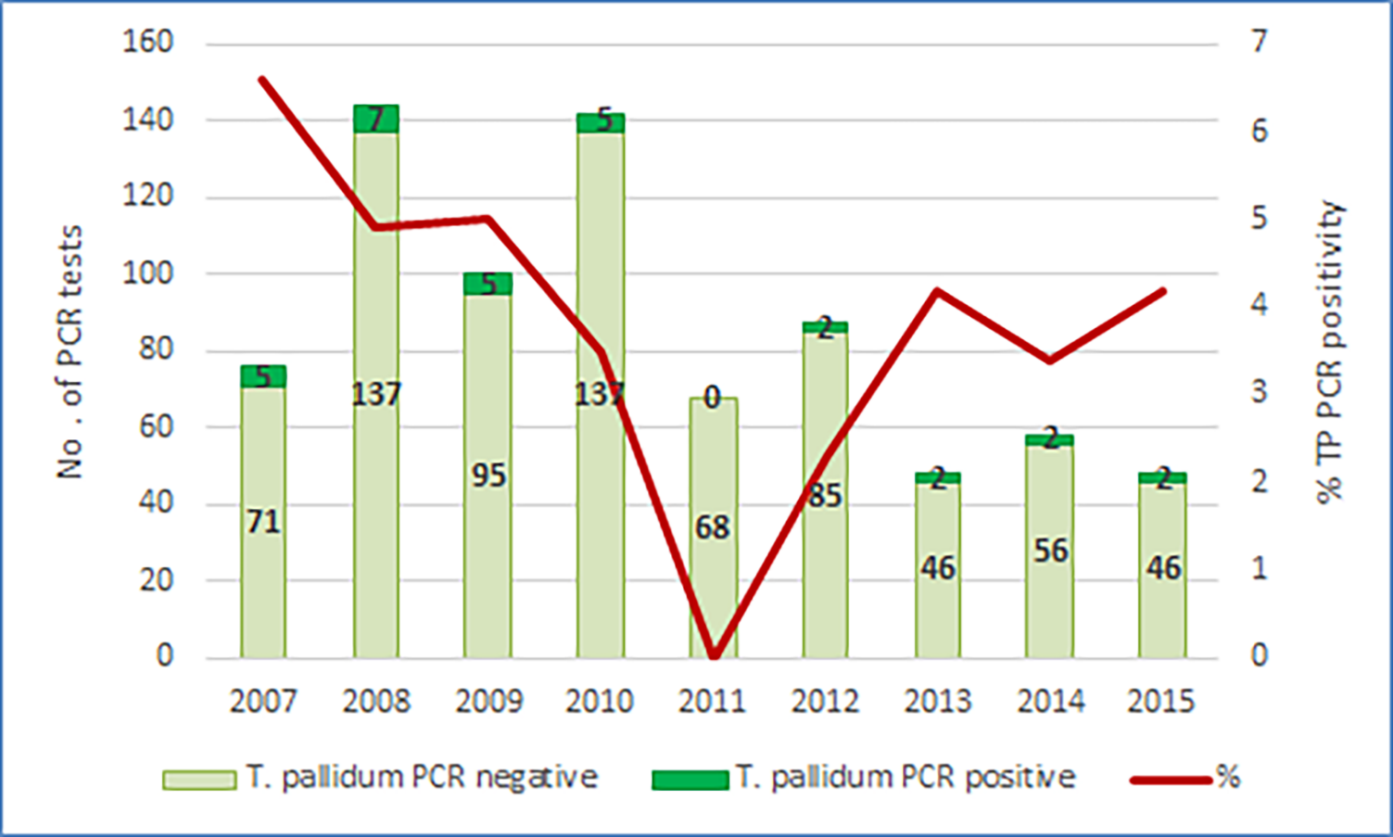
Number of *T*. *pallidum* PCR positive and negative genital ulcers, with the prevalence trend for PCR-confirmed syphilitic ulcers (red line), 2007–2015 (n = 771).

Laboratory results for genital and blood specimens were available for all participants. Of 771 genital swab specimens, 503 (65.2%) had a PCR-detectable ulcer-derived STI pathogen. The overall prevalence of pathogens (Table 3) was as follows: HSV 468 (60.7%); TP 30 (3.9%); CT L1-3 7 (0.9%); HD 4 (0.5%). The relative prevalence of the most commonly implicated pathogen, HSV, ranged from 48% (24/50) in 2015 to 74.5% (76/102) in 2009 (Fig 1). All HSV-related ulceration was caused by HSV-2; there were no HSV-1 ulcerative lesions. The prevalence of TP causing primary syphilis varied from 0% (0/68) in 2011 to 6.6% (5/71) in 2007 (Fig 2). No cases of granuloma inguinale were detected. Six patients (0.8%) had mixed ulcer aetiologies detected by PCR; all were co-infected with HSV and a bacterial pathogen: TP (3), CT L1-L3 (2), and HD (1). The last case of chancroid was detected in 2009, and the last case of lymphogranuloma venereum in 2012. An aetiological agent was not identified by PCR in the ulcers of 270 (34.8%) patients.

**Table 3:** Microbial aetiology of GUD at a community healthcare centre in Johannesburg, 2007–2015.

Pathogen	Prevalence (95% CI)	N = 771
Herpes simplex virus	60.7% (57.2–64.1)	468
*Treponema pallidum*	3.9% (2.7–5.5)	30
*Chlamydia trachomatis* L1-L3 (Lymphogranuloma venereum)	0.9% (0.4–1.9)	7
*Haemophilus ducreyi*	0.5% (0.2–1.4)	4
*Klebsiella granulomatis*	0%	0
Mixed aetiology (HSV-2 + bacterial pathogen)	0.8% (0.4–1.7)	6
No STI pathogen	34.8% (31.5–38.2)	268

### Seroprevalence of co-infections

**Fig 3:**
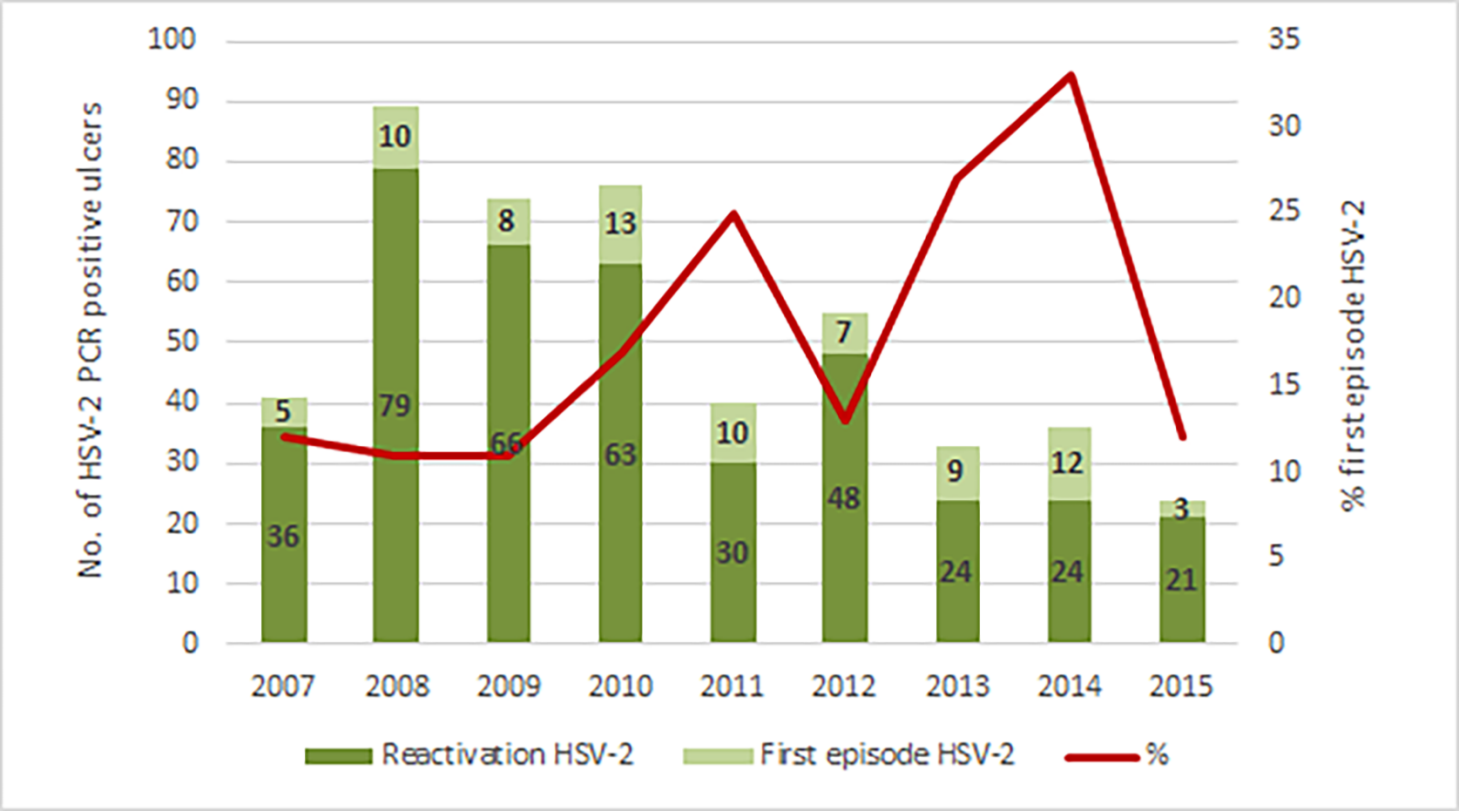
Number of reactivation and first-episode HSV-2 among patients with ulcer-detected HSV (n = 468), with prevalence trend for first-episode HSV-2 (red line), 2007–2015.

Seroprevalence rates were as follows: HIV 61.7%; HSV-2 80.2% and RPR 5.8%. Among patients with genital herpes, 83.5% (391/468) were both HSV-2 PCR and HSV-2 serology positive, representing reactivation disease. The average prevalence of first-episode HSV-2, where patients with ulcer-detected HSV-2 tested negative on HSV-2 serology, was 16.5% (77/468); and ranged from 11% in 2008–2009 to 33% in 2014 (**Fig 3**). Among 30 patients with primary syphilis confirmed by ulcer PCR, the sensitivity of RPR serology was 60% (18/30). Forty-percent (12/30) of patients with laboratory-confirmed primary syphilis were RPR negative; of these the majority, 75% (9/12), were HIV-infected. Most patients with RPR-positive primary syphilis were HIV-uninfected (12/18; 66.7%). HIV co-infection rates among GUD patients ranged from 72.6% (55/76) in 2007 to 44.8% in 2014 (26/58).

### Trends and associations of GUD aetiologies

There was a strong association between GUD pathogen detection and HIV seropositivity (p < 0.001); 68% of cases caused by HSV-2 were co-infected with HIV. Overall, HIV-uninfected patients, as determined by serological testing, were more likely than those who were HIV-infected to have GUD of unknown aetiology by PCR (OR 1.87, 95% CI = 1.38–2.52, p < 0.001), and less likely to present with HSV-associated ulceration (OR = 0.50; 95% CI = 0.37–0.67; p < 0.001).

Logistic regression analysis for association of no ulcer aetiology and HSV by gender (in a population excluding those with other microbial causes of GUD), showed that males were less likely than females to have no ulcer aetiology (0R = 0.72; 95% CI = 0.52–0.99; p < 0.001). There was no significant difference in the likelihood of males presenting with HSV-associated GUD than females (OR = 1.19; 95% CI = 0.87–1.63; p = 0.268).

**Fig 4:**
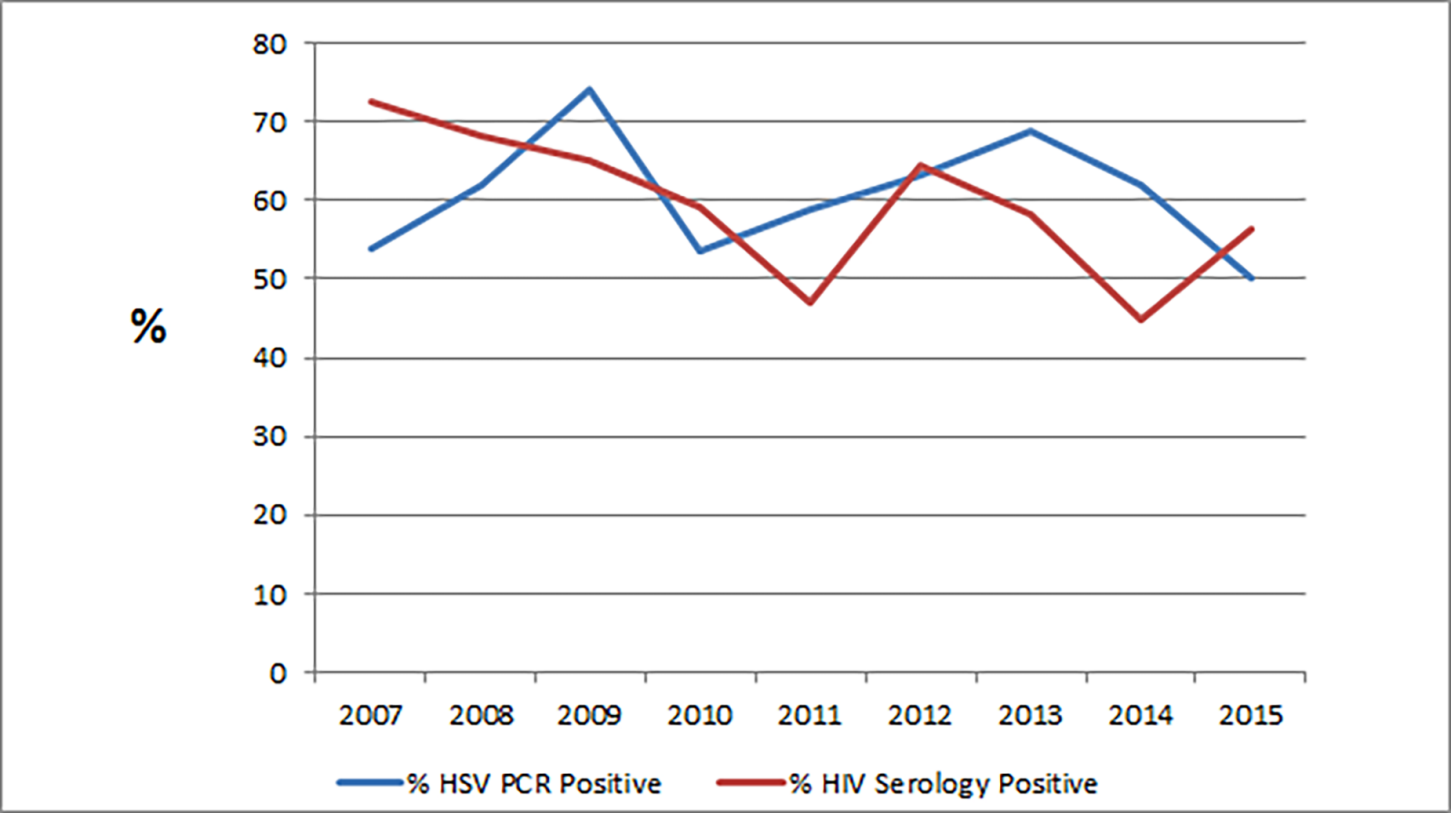
Trends in HSV and HIV prevalence in GUD, Johannesburg 2007–2015 (n = 771).

There was a significant decline in the relative prevalence of PCR-detectable ulcer-derived HSV over time, predominantly from 2013–2015 (p-value for trend 2007–2015 = 0.023); and a statistically insignificant trend towards a decrease in the HIV seropositivity rate (p-value for trend 2007–2015 = 0.209) (Fig 4). However, in an analysis which only included patients with a laboratory-proven ulcer aetiology, the decrease in the relative prevalence of ulcer-derived HSV over time was not statistically significant (p = 0.449; p-value for trend 2007–2015 = 0.297). When stratified by HIV serostatus, there was no significant change in prevalence trend of HSV over time in the HIV-infected group (p-value for trend = 0.293); however, there was an insignificant downward trend in the relative prevalence of HSV ulceration (predominantly between 2013–2015) in the HIV-uninfected population (p-value for trend = 0.063).

## Discussion

We present trends in the relative prevalence of GUD aetiologies over a nine-year period. Our data reveal that, in keeping with findings from other surveillance studies, HSV-2 has remained the leading cause of pathogen-detectable GUD at Alexandra Health Centre [11], and this supports the use of anti-viral therapy in the syndromic management guidelines since 2008. The WHO recommends inclusion of anti-herpes therapy in GUD treatment algorithms if HSV is responsible for 30% or more of GUD.[7] A change in epidemiology to HSV-1 has not been observed, as is the case in high-income countries where genital HSV-1 has been identified as the leading cause of incident GUD, especially in young heterosexual women and MSM.[13] This change in Western Europe and USA has been attributed to a rise in socio-economic conditions with a corresponding reduction in childhood acquisition of HSV-1, as well as a transformation in sexual practices with a preference for oro-genital sexual intercourse. Our data support anecdotal findings that oro-genital sex is not a popular practice among the predominantly heterosexual populations accessing STI services at the primary healthcare centre.

Approximately 80% of HSV ulceration in our study represented reactivated disease and there appeared to be a decline in the relative prevalence of HSV-related GUD at Alexandra Heath Centre over the time period studied. There are several factors that may have contributed to this trend. Since 2004, there has been increased access to anti-retroviral therapy (ART) for HIV-infected individuals and a scale-up of ART coverage in the South African public healthcare sector. Anti-retroviral therapy is not associated with reduced HSV shedding [14]; however, it may lead to a decrease in clinically apparent HSV reactivation.[15] Recent efforts to increase HIV testing may have led to earlier HIV diagnoses and initiation of therapy at higher CD4+ T-lymphocyte counts, resulting in less immunosuppression and reduced HSV-2 shedding in newly diagnosed HIV-infected patients. Additionally, male medical circumcision, which is a component of the national HIV/AIDS prevention package, is associated with a reduction in incident HSV ulceration.[16] However, the trend in decline of HSV over time was not statistically significant in the subgroup of patients with a confirmed ulcer-derived STI pathogen. It is possible that the sample-collecting technique of the nurses, clinical decision-making on ulcer sampling, and consequently specimen adequacy may have altered over time and maybe a contributory factor in the relative increase in ulcers without a detectable pathogen.

The sensitivity of RPR in suspected primary syphilis is expected to be 70–80%.[17] We hypothesize that it was lower in our patients with ulcer-proven *Treponema pallidum* aetiology because a small proportion may have presented with very early primary infection prior to seroconversion. HIV infection may also lead to an aberrant serological response, such as a delay in the development of antibody to syphilis infection. It has been noted that initial non-treponemal serology may be negative more often in primary syphilis than in other disease stages, particularly in populations with relatively high rates of HIV co-infection.[18] Increased false-negative non-treponemal serology due to prozone phenomenon has also been described.[19] Primary syphilis and LGV appear to be relatively uncommon causes of GUD among our patients. However, these pathogens are increasingly being recognised among MSM in Western Europe, where there has been a resurgence of syphilis as the predominant cause of GUD, and a rising incidence of LGV proctitis. [20, 21] An important component of future STI surveillance strategies will be the incorporation of aetiological monitoring in key populations.

From 2000 onwards, following the successful implementation of syndromic management as recommended by the WHO, the proportion of GUD caused by *Haemophilus ducreyi* has declined substantially worldwide.[8] Accordingly, chancroid, a predominant cause of GUD in South Africa in the 1980s and early 1990s (and, previously, responsible for up to 70% of genital ulceration), is now detected on a sporadic basis only. Donovanosis has a limited geographical distribution and is very rarely a cause of GUD aetiology in South Africa. Previous cases have been mostly located in rural Kwa Zulu Natal, and may have facilitated the acquisition and spread of the HIV epidemic in these areas.[22] The condition may be missed as diagnosis is made using relatively insensitive phenotypic methods. Commercial PCR assays have not yet been developed and validated for clinical use.

Approximately 35% of patients presented with ulcers that could not be attributed to a definitive STI pathogen. This is in keeping worldwide epidemiological trends and data from other sub-Saharan African countries.[23] In Kenya, among men enrolled in a male circumcision trial, GUD of unknown aetiology was associated with a higher bacterial diversity as determined by ribosomal RNA sequencing.[24] These included mostly anaerobic species, some of which have histolytic properties and are associated with enhancing epithelial disruptions. In our survey, GUD of unknown aetiology was relatively more common in HIV-seronegative patients. Acute HIV-associated ulceration may also be a cause. Among South African men enrolled in a clinical trial of acyclovir treatment for genital ulcer disease, acute HIV infection was associated with an almost eight times greater likelihood of having genital ulcers of unknown aetiology compared with herpetic ulcers.[2] In the same trial, over 45% of HIV-infected men were found to have detectable HIV-1 RNA in ulcers, and those with ulcers of unknown aetiology were more likely to have HIV-1 lesional shedding than those with HSV-2 ulceration.[3] Other possible causes include non-infectious conditions such as genital trauma and Behçet’s syndrome [25] or, rarely, malignancies such as squamous cell carcinoma.[26–29] Further investigation and research are needed to properly elucidate the aetiology, and should include biopsy and histopathology, as well as next-generation sequencing of ulcer material, particularly for persistent or recurrent ulceration unresponsive to standard syndromic management.

The high HIV seroprevalence rate among GUD patients reflects the epidemiological synergy between STIs and HIV, and biological plausibility for co-infection due to common sexual behaviour and transmissibility. Factors that contribute to this synergy include disruption of genital mucosal epithelium and recruitment of inflammatory HIV target cells.[30] It has been demonstrated that genital ulcer disease and HSV-2 seropositivity are associated with HIV acquisition and seroconversion, and an increased HIV viral load.[31] The risk of HIV transmission is estimated to be highest during the initial stage of early HIV infection.[32] This underscores the importance of linkage to universal HIV testing and treatment for GUD patients at primary healthcare level. These data support the 2016 universal test and treat strategy formally adopted by the South African National Department of Health, in accordance with WHO evidence-based guidelines, whereby all HIV-infected individuals are offered anti-retroviral therapy (ART) regardless of CD4+ T-lymphocyte count.

Strengths of the study include the large number of participants enrolled over a nine-year surveillance period. Seasonal variability may occur as there is a migrant male labour population that travel home to rural areas in other provinces over the December holidays, therefore sexually transmitted infections are typically seen more frequently in January and February of each year. We controlled for this by undertaking surveillance and collecting samples during the same months every year during the study period. This should have eliminated any bias due to seasonal variation in prevalence. Limitations include missing demographic and clinical data for a proportion of the participants. The lack of additional clinical or diagnostic information for patients with GUD of unknown aetiology is an important limitation that requires further investigation. Future GUD aetiological prevalence surveys should collect information on the proportion of HIV-infected individuals who are adherent on ART, as well as the proportion of male participants who have been medically circumcised. The relatively small numbers of participants enrolled in the latter years of surveillance may make prevalence estimates unstable from one year to the next. Additionally, the findings from this single-site surveillance study may not necessarily be generalizable to GUD patients presenting to primary healthcare facilities in other regions of the country.

## Conclusion

Herpes simplex virus remains the leading cause of pathogen-detectable GUD in South Africa, justifying the continued use of acyclovir in syndromic management. Aetiological trends reveal a decline in the relative prevalence of HSV-2 associated ulceration, possibly resulting from increased uptake of HIV anti-retroviral therapy and HIV preventive strategies in the public health sector. The aetiology of ulcers without detectable STI pathogens in a significant proportion of patients requires further research. The prevalence of HIV co-infection among GUD patients is high, underlining the importance of linkage to universal HIV testing and treatment for these patients in primary healthcare settings.

## Supporting information

S1 Dataset (XLSX)
